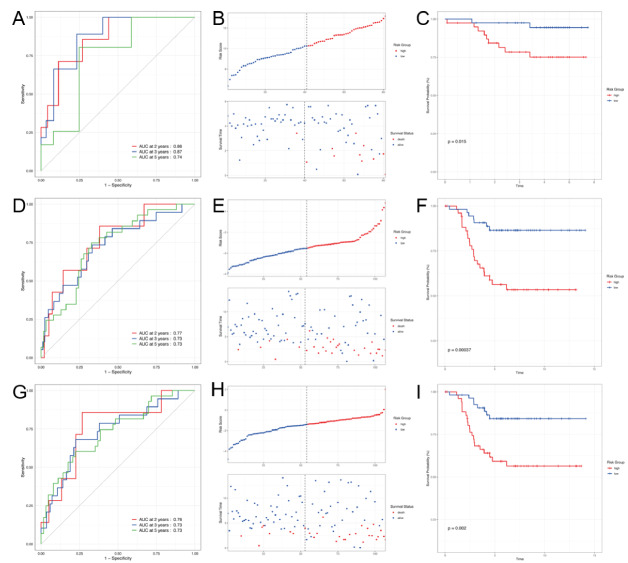# Identification of the new molecular subtypes related to inflammation in breast cancer

**DOI:** 10.1097/MD.0000000000038146

**Published:** 2024-05-10

**Authors:** Ke Yu, Chi Xu, Feng Wang, Hua Wang

**Affiliations:** a Department of Breast and Thyroid Surgery, Affiliated Hospital of Nantong University, Nantong, Jiangsu Province, China; b Department of Breast and Thyroid Surgery, Clinical Medicine, Medical College, Nantong University, Nantong, Jiangsu Province, China; c Department of Gastroenterology, Affiliated of Nantong University, Nantong, Jiangsu Province, China; d Department of Laboratory Medicine, Affiliated Hospital of Nantong University, Nantong, Jiangsu Province, China.

**Keywords:** breast cancer, inflammatory response, molecular subtype, tumor microenvironment (TME)

## Abstract

Breast cancer is a prevalent ailment among women, and the inflammatory response plays a crucial role in the management and prediction of breast cancer (BRCA). However, the new subtypes based on inflammation in BRCA research are still undefined. The databases including The Cancer Genome Atlas and gene expression omnibus were utilized to gather clinical data and somatic mutation information for approximately 1069 BRCA patients. Through Consensus Clustering, novel subtypes linked to inflammation were identified. A comparative analysis was conducted on the prognosis, and immune cell infiltration, and somatic mutation of the new subtypes. Additionally, an investigation into drug therapy and immunotherapy was conducted to distinguish high-risk individuals from low-risk ones. The findings of this investigation proposed the categorization of BRCA into innovative subtypes predicated on the inflammatory response and 6 key genes were a meaningful approach. Specifically, the low-, medium-, and high-inflammation subtypes exhibited varying degrees of association with clinicopathological features, tumor microenvironment, and prognosis. Notably, the high-inflammation subtype was characterized by a strong correlation with immunosuppressive microenvironments and a higher frequency of somatic mutations, which was an indication of poorer health. This study revealed that a brand-new classification could throw new light on the effective prognosis. The integration of multiple key genes was a new characterization that could promote more immunotherapy strategies and contribute to predicting the efficacy of the chemotherapeutic drugs.

## 1. Introduction

Inflammation is a defensive reaction that encompasses immune cells, blood vessels, and molecular mediators.^[1]^ It constitutes a pivotal element of the host’s defense mechanism, safeguarding the body against harm and infection.^[2]^ Nevertheless, persistent or controlled inflammation can result in tissue impairment and malfunction, frequently contributing to the pathogenesis of numerous ailments.^[3]^ During the inflammatory process, numerous cytokines and chemicals are released by damaged cells, potentially inducing aberrant cellular growth, and impeding the immune system’s capacity to target cancerous cells, thereby facilitating the propagation and migration of malignant cells.^[4]^

Nowadays, some research revealed that different inflammation responses may have different effects on the tumor growth.^[5]^ Ulcerative colitis, for instance, is an inflammatory bowel disease that may increase colon cancer risk. The inflammatory response is indispensable in the pathogenesis of colon cancer.^[6–8]^

Breast cancer is a common malignant tumor, which presents a significant hazard to women’s health.^[9,10]^ The current treatment of breast cancer is presently divided into different subtypes based on hormone receptor (HR) and human epidermal growth factor receptor 2 (HER2).^[11]^ However, recent studies have underscored the importance of the tumor microenvironment (TME) in the diagnosis, treatment, and prognosis of breast cancer within the academic community.^[12]^ The TME is characterized by the release of various signaling molecules by breast cancer cells, and inhibition of immune cells in the TME can facilitate tumor growth and metastasis.^[13]^ As an illustration, the secretion of matrix metalloproteinases and interleukin-8 by fibroblasts and inflammatory cells surrounding tumors can alter the matrix structure within the tumor microenvironment, thereby promoting tumor growth.^[14]^

Given this background, it is hypothesized that the classification of breast cancer subtypes based on the inflammatory response could correlate with distinctive clinical characteristics, prognostic outcomes, TME conditions, and therapeutic responses. This study aims to categorize breast cancer (BRCA) into various subtypes predicated on inflammatory response, evaluate the prognostic significance of these subtypes and their correlation with the TME, develop and validate a relevant prognostic model, and analyze the efficacy of common chemotherapeutic agents in breast cancer treatment. This approach seeks to underscore the pivotal role of inflammation in breast cancer research and its potential to inform tailored therapeutic strategies.

## 2. Materials and methods

### 2.1. Data gathering and processing

This study utilized a dataset comprising 1069 patients diagnosed with BRCA, sourced from the UCSC Xena website (https://xenabrowser.net/datapages/), which included both their corresponding somatic mutation and clinical information. Additionally, we employed data from the Gene Expression Omnibus (GEO) database (GSE48390, GSE58812, GSE103091) as a means of verifying our findings. Molecular signatures database is a repository of hallmark gene sets that have been selected to characterize the inflammatory response (https://www.gsea-msigdb.org/gsea/msigdb/cards/HALLMARK_INFLAMMATORY_RESPONSE.html). To assess the efficacy of immune checkpoint inhibitor, we employed an external validation dataset, namely IMvigor210, which was procured from IMvigor210CoreBiologies (http://research-pub.gene.com/IMvigor210CoreBiologies/).

The data utilized in our articles were made publicly accessible by the providers, in compliance with their individual publication mandates and data accessibility protocols.

### 2.2. Identification of molecular subtypes based on inflammation

A consensus clustering approach was implemented in R using the “ConsensusClusterPlus” package to identify molecular subtypes associated with inflammation.^[15]^ The optimal cluster numbers were subsequently determined within the range of k = 1 to 10. This process was repeated multiple times for the validity and stability of the outcome. Finally, the pheatmap function in R was used to generate a cluster map.

### 2.3. Signal sample gene set enrichment analysis

To assess the inflammation response of BRCA samples, we used the single-sample gene-set enrichment analysis (ssGSEA) analysis and completed the final data with the aid of the “GSVA” and “GSEABase” packages in R.^[16]^ Using gene set enrichment analysis, it was possible to determine the gene signature for inflammation response.

### 2.4. Principal component analysis

The study used principal component analysis to examine the extent of differentiation among various subtypes of inflammation. Gene names were imported alongside corresponding sample data and expression levels. The analysis was subsequently executed using the “tinyarray” package’s draw_pca function, and the outcomes were graphically represented by the “ggplot2” package in R.

### 2.5. Immune infiltration analysis

BRCA samples were scored using the “ESTIMATE” algorithm of the R package.^[17]^ The calculation of the ESTIMATE score was predicated upon the stromal and immune scores, which exhibited an inverse correlation with tumor purity. We further explored if inflammatory subtypes are meaningful in breast cancer by comparing the ESTIMATE scores of the 3 subtypes and the purity of the tumor.

The abundance of TME 28 immune cells was conducted through ssGSEA including effector memory CD8 T cell, myeloid-derived suppressor cells (MDSC), type 1 T helper cell, activated dendritic cell, immune B cell, T follicular helper cell, activated B cell, regulatory T cell, activated CD 8 T cell, activated CD4 T cell, natural killer T cell, gamma delta T cell, macrophage, effector memory CD4 T cell, monocyte, central memory CD4 T cell, natural killer cell, central memory CD8 T cell, mast cell, Type 2 T helper cell, plasmacytoid dendritic cell, immature dendritic cell, type 17 T helper cell, CD 56bright killer cell, eosinophil, memory B cell, CD 56dim natural killer cell, neutrophil.^[18,19]^

In regard to the immune checkpoint, a comparison was made between 8 common checkpoints (PD-L1, CTLA4, IDO1, LAG3, PD1, PDCD1LG2, TIGIT, and TIM-3) across various subtypes.^[20]^ The draw_boxplot function was utilized to demonstrate the association between immune checkpoints and 3 distinct inflammatory subtypes.

### 2.6. Estimation of the immune activity about 3 inflammatory subtypes

For an antitumor immune response to effectively eliminate tumor cells, it is essential to initiate and allow progressive events to take place continuously and iteratively. A cancer immune cycle entails these steps. The immune cycle typically comprises 7 distinct stages, with varying levels of inflammatory responses exhibited at each stage. To visually represent the activity of different inflammatory subtypes across these stages, the “ggradar” function was employed to generate an immune cycle radar map.

Additionally, a selection of twenty negative regulatory immune genes was identified from the The cancer genome atlas (TCGA) database, and their distribution across the various inflammatory subtypes was analyzed using the pheatmap function to create heat maps.

### 2.7. Reaction ontology (REACTOME), Kyoto encyclopedia of genes and genomes (KEGG), and gene ontology (GO) enrichment analysis

GO enrichment analysis (http://geneontology.org/), KEGG (Kyoto encyclopedia of genes and genomes) (https://www.genome.jp/kegg/), and REACTOME (https://reactome.org/) pathway analysis were all used in the GSEA analysis to identify overlaps between genes. With GO analysis, biological information can be understood holistically, enabling identification of the functions and interconnections among a wide range of organisms. The KEGG pathway analysis, on the other hand, identified the biological pathways that were linked to the DEGs. Both the standards of GO and KEGG analyses were cut off at a *P*-value of .05 or less. Finally, the REACTOME pathway database serves as a pathway analysis tool that effectively mitigates the challenges associated with multi-omics and comparative pathway analysis. The goal is to provide visual, interpretable analysis of each pathway to support basic research, clinical studies, genomic analysis, modeling, systems biology, and more.

### 2.8. Somatic mutation analysis

The TCGAbiolinks tool was utilized to procure somatic mutation data in“maf” format for every BRCA sample.^[21]^ Using the R software’s “maftools” function, waterfall charts were generated, which allowed the altered genes of the 3 subtypes and unusual signal pathways to be visualized and summarized.^[22]^ After calculating tumor mutation burdens for each individual, comparisons were made between groups at high- and low-risk.

### 2.9. Modeling and verification of prognostic risk

Initially, the identification of genes significantly associated with inflammatory response was accomplished through univariate Cox proportional-hazards model regression analysis. Subsequently, the hub genes were screened using the random forest algorithm and the least absolute shrinkage and selection operator (LASSO) algorithm. A regression analysis using the LASSO Cox method was conducted using the “glmnet” package in R.^[23]^ As a final step, Random Forest classifiers were developed using the “randomForestSRC” R package (version 3.2.1).^[24]^

The present study identified a series of hub genes through sequential application of 2 algorithms. To investigate potential predictive markers, a multivariate Cox analysis was performed, followed by the development of a prognostic model. The clinical prognostic characteristics were determined by the risk score based on the aforementioned key genes. Kaplan–Meier analysis was used for 2 risk groups using “Survminer” and “Survival” packages, and the results were compared.^[25,26]^

The receiver operating characteristic (ROC) curve analysis was used to assess how well the risk model predicts patients over a 2, 3, and 5-year period. In addition, 3 data sets (GSE48390, GSE58812, and GSE103091) were selected from the GEO database for model verification.

### 2.10. Evaluation of drug sensitivity based on the signature

The IC50 determines how sensitive a drug is to a particular drug by determining what concentration of the drug is required to inhibit the growth of tumor cells by 50%, with a lower value indicating a stronger response. To assess the potential of this risk model in guiding chemotherapy for BRCA patients in the TCGA cohort, the “OncoPredict” package was utilized to calculate the IC50 values of commonly used chemotherapeutic treatments.^[27]^ The selection of drugs was based on the recommendations provided by the AJCC guidelines such as Epirubicin, Docetaxel, Cyclophosphamide, Cisplatin, and Paclitaxel for the treatment of BRCA. An analysis of IC50s was conducted with the t-test, and the results are presented in the form of a box plot showing the distinction between those at high and low risk. To enhance the precision of prognostic assessment for immunotherapy, a novel prognostic evaluation feature, namely risk score, was utilized. The IMvigor210 cohort, which received anti-PD-L1 immunotherapy, was retrieved from IMvigor210CoreBiologies.^[28]^ The significance of the inflammatory risk score was further validated by comparing the effectiveness of immune checkpoint inhibitors across various subtypes. The results of the analysis were visualized through the utilization of the “ggplot” and “ggboxplot” functions.

### 2.11. Statistical analysis

R version 4.2.2 was used for the statistical analyses. An acceptable level of statistical significance is *P* < .05. In the comparison of continuous variables that followed a normal distribution between 2 groups, *t*-tests and Wilcoxon tests were utilized. Several groups were compared using an ANOVA. An analysis of survival curves was performed using the Kaplan–Meier method and the log-rank test. The model’s independent prognostic value was verified through multivariate Cox regression. An analysis of the time-dependent ROC curve was conducted to determine whether the risk gene signature model has a prognostic value.

## 3. Results

### 3.1. Consensus clustering classified sample data based on inflammation

**Figure 1.**
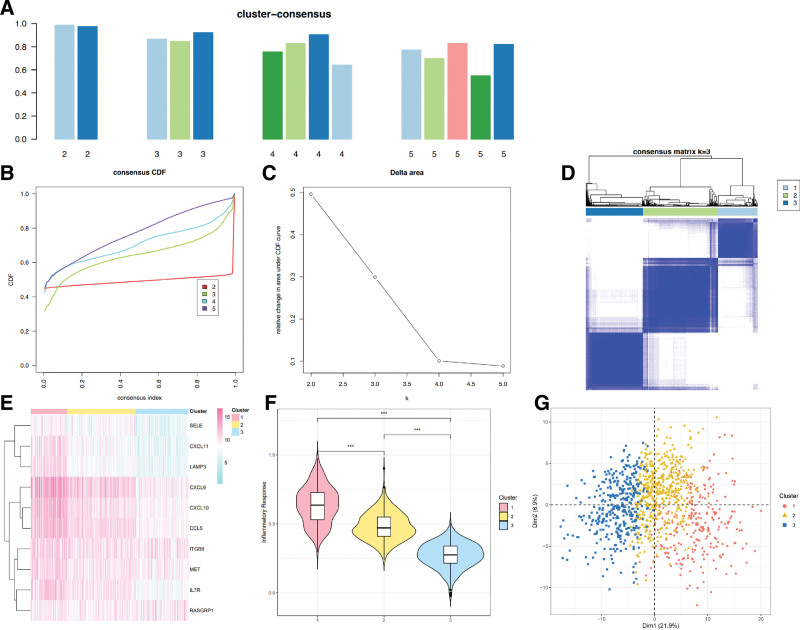
Classification of BRCA samples based on inflammation. (A) Proportion of sample size in different clusters. (B) Graph of consensus clustering in Delta. (C) Clustering heatmap (K = 3). (D) The expression levels of 10 inflammatory genes in 3 groups depicted by heat map; the color pink symbolizes high expression, and the color blue denotes low expression. (E) Violin plot indicates the distinctions among 3 groups. (F and G) Plot of PCA. BRCA = breast cancer, PCA = principal component analysis.

Inflammation-related genes were obtained using gene set enrichment analysis, which was subsequently utilized in identifying the BRCA inflammation-based clusters via Consensus Clustering. Subsequently, the TCGA cohort was partitioned into 3 clusters (Fig. 1A, B, and C). Significantly, there was a variation in the expression of inflammatory genes across the distinct clusters, with cluster 1 (C1) displaying the highest levels, cluster 2 (C2) showing medium levels, and cluster 3 (C3) exhibiting the lowest expression (Fig. 1D).

Additionally, the ssGSEA method was utilized to assess the inflammatory response of the 3 clusters. The findings demonstrated that patients in C1 exhibited the highest score, while those in C3 had the lowest score (Fig. 1E). As a result, we designated C3 as an inflammation-low subtype, C1 as an inflammation-high subtype, and C2 as an inflammation-media subtype. Following that, principal component analysis was conducted to assess the transcriptional patterns among the 3 inflammatory subtypes (Fig. 1F–G) (Fig. S1, Supplemental Digital Content, http://links.lww.com/MD/M493).

### 3.2. The expression levels of ER, PR, and HER2 across various subtypes

**Figure 2.**
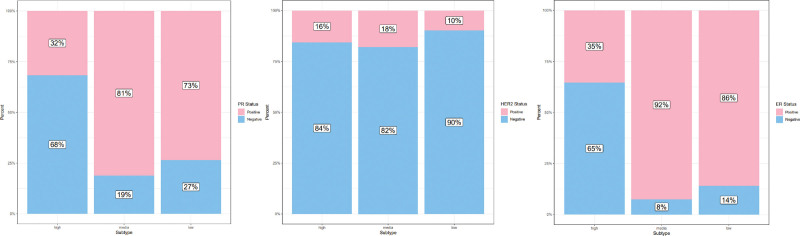
The variation in hormone receptor expression across 3 distinct subtypes. A ER, HER2, PR status among these subtypes. ER = estrogen receptor, HER2 = human epidermal growth factor receptor 2, PR = progesterone receptor.

Prior research has demonstrated the significant involvement of inflammatory responses in the breast cancer tumors. This study presented a comparative analysis of the clinicopathologic characteristics of 3 distinct subtypes. Our findings indicate that the high-inflammation subtype exhibited predominantly negative estrogen receptor (ER) and progesterone receptor (PR) statuses, whereas the low and medium subtypes displayed a greater prevalence of positive ER and PR statuses (Fig. 2A).

### 3.3. Comparison of the immune infiltration in TME

**Figure 3.**
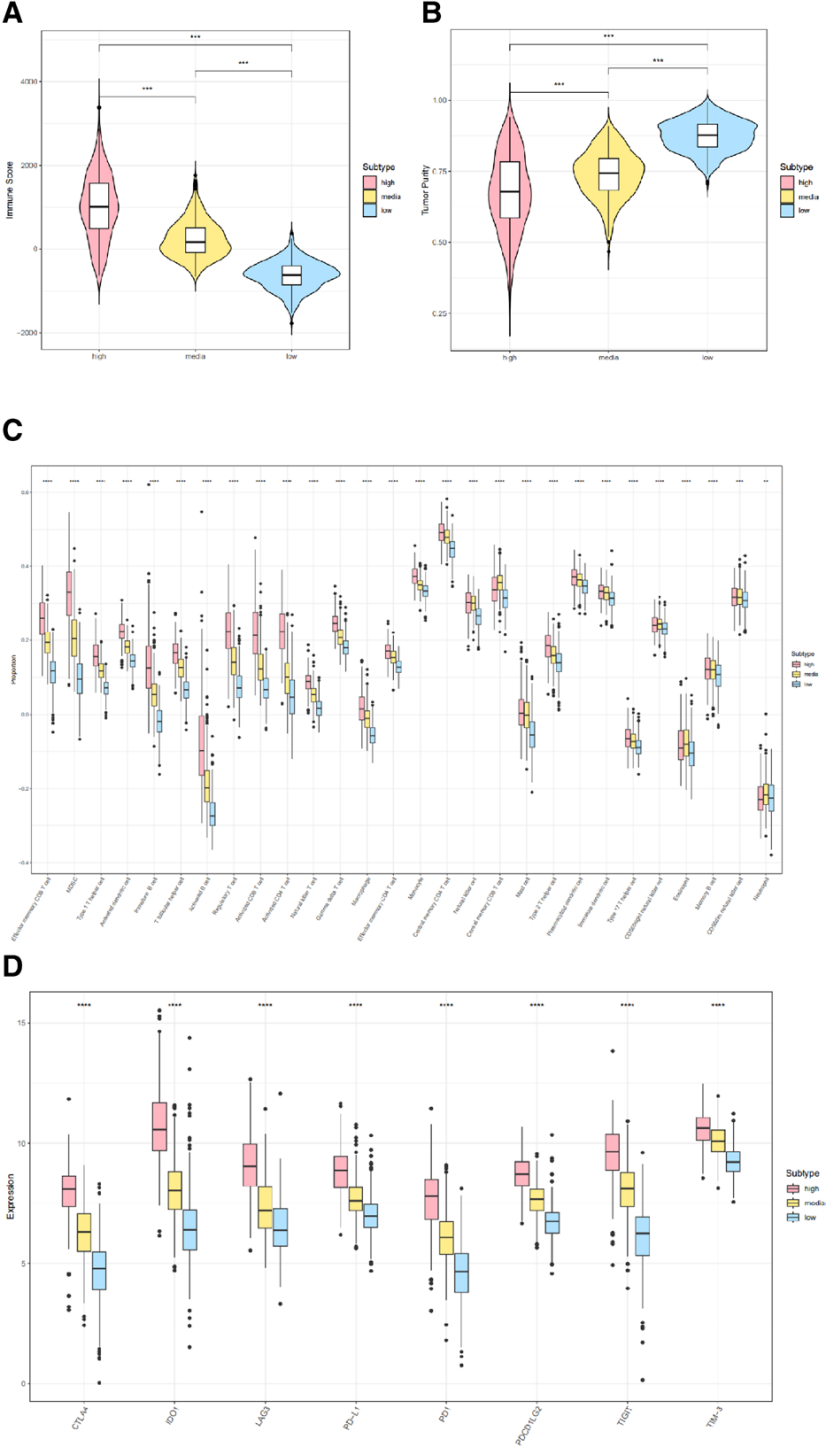
Distinct tumor microenvironments correlated with inflammatory subtypes. (A) Violin plot showing immune score. (B) Violin plot presenting tumor purity score. (C) Boxplots representing 28 immune cells infiltration in 3 subtypes. (D) Boxplots representing immune checkpoints in different inflammation subtypes.

TME, particularly its immune cells, is greatly influenced by inflammation. To investigate the TME compositions across various subtypes, we conducted an analysis. The outcomes indicated that the cohort exhibiting elevated levels of inflammation demonstrated the most robust immune response, while those with moderate inflammation exhibited a moderately strong response, and those with low inflammation exhibited the weakest response (Fig. 3A), while tumor purity exhibited a gradual increase (Fig. 3B). There appeared to be a positive correlation between higher levels of inflammation infiltration and increased immune cell numbers.

To further assess the immune heterogeneity among these subtypes, we employed the ssGSEA method. Figure 3C presents a comprehensive overview of 28 distinct immune cell infiltrations. Notably, patients belonging to the high inflammatory subtype exhibited elevated expression levels of most immunosuppressive cells, particularly Regulatory T cells and MDSC. Conversely, the expression levels of central memory CD8 T cells, CD56bright natural killer cells, Eosinophils, and Neutrophils were comparatively lower in this subtype than in the other 2 subtypes.

Furthermore, the inflammation-high subtype exhibited predominantly elevated expression levels of immune checkpoints, while the inflammation-low subtype demonstrated the lowest expression levels (Fig. 3D). These observations elucidated the potential role of immunosuppressive cells, activated CD4+ T cells, and heightened expression of immune checkpoints in driving the immunosuppressive microenvironment of the inflammation-high subtype.

### 3.4. The assessment of immune activity against cancer in various subtypes of inflammation

**Figure 4.**
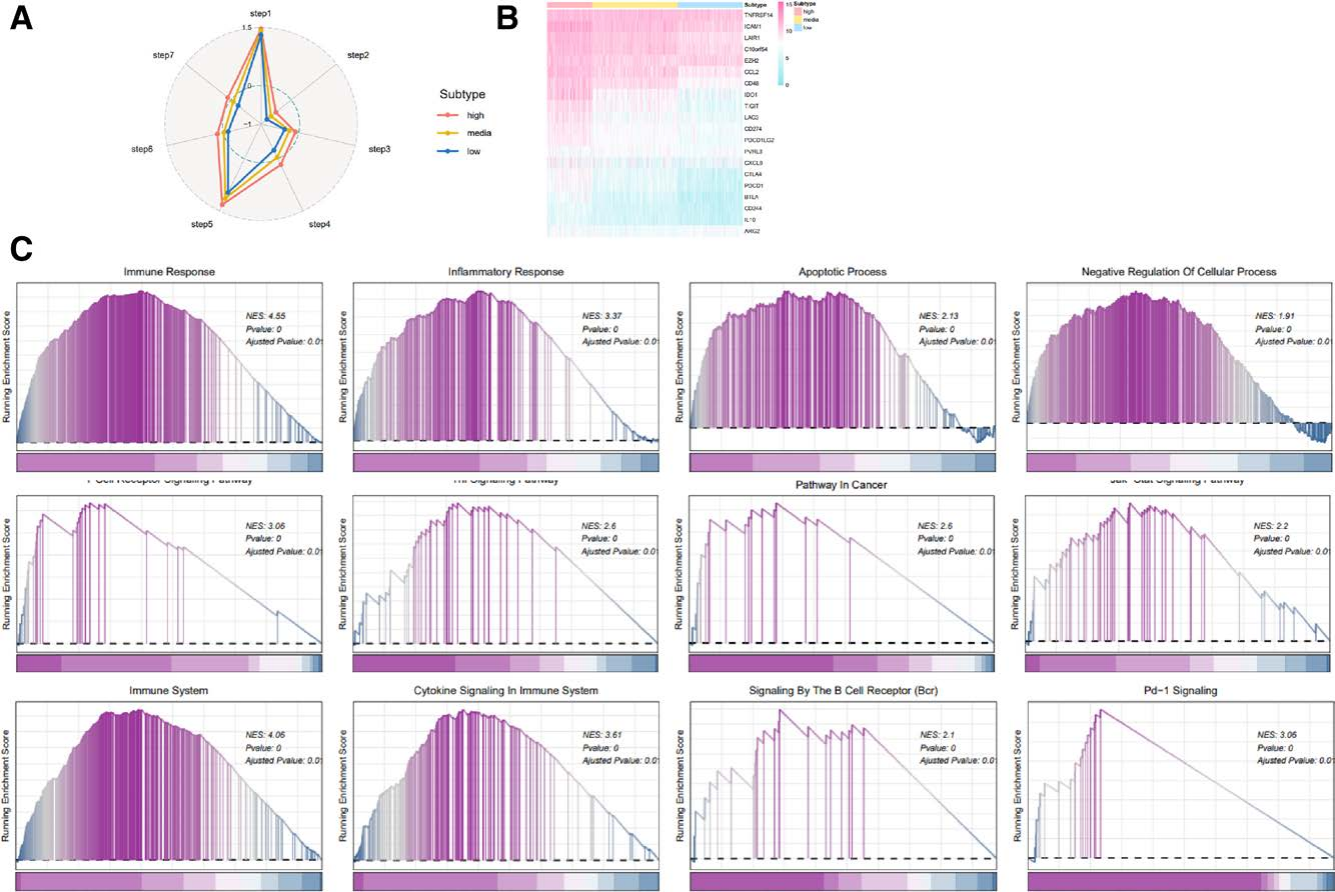
Evaluation of the activity of inflammatory subtypes against cancer. (A) Cancer-immune cycle of 7 steps. (B) Heatmap displaying expression gene functioning as negative regulation. (C) Biological pathways revealed through GSEA analysis. GSEA = gene-set enrichment analysis.

The concept of antitumor immunity can be understood as a series of 7 consecutive processes, collectively known as the “cancer-immunity cycle.” This study employed TIP, a web service specifically developed for the purpose of determining tumor immunophenotype profiling, to assess the anticancer immunological function of the cancer-immunity cycle across 3 distinct subtypes. Our study suggested that the high-inflammation subtype exhibited significant activity in all steps of the cycle, including step 1 (antigen release from the tumor), step 2 (tumor antigen presentation), step 3 (priming and activation), and step 4 (trafficking of T cells in tumors), and step 5 (immune cell infiltration into the tumor), and step 6 (recognition of cancer cells by T cells), and step 7 (killing of cancer cells) (Fig. 4A).

The top 20 genes were chosen to construct a heat map, which revealed that genes that regulate the immune system negatively were predominantly expressed at high levels in the inflammation-high subtype, followed by the inflammation-medium and -low subtype (Fig. 4B). This suggests that the immunosuppressive microenvironment in the high inflammation subtype may frequently result in an unfavorable prognosis for breast cancer.

Furthermore, we analyzed potential pathways linked to the inflammatory subtypes. The results showed that the high inflammatory subtype has significant effects on several pathways such as immune response, inflammatory response, apoptotic process, negative regulation of cellular process, T cell receptor signaling pathway, Tnf signaling pathway, Pd-L1 expression, and Pd-1 checkpoint pathway in cancer, Jak-Stat signaling pathway, immune system, cytokine signaling in immune system, signaling by the B cell receptor, and PD-1 signaling were significantly positively regulated, while almost all of these pathways played an important role in negative regulation (Fig. 4C).

The aforementioned results indicate that individuals with the inflammation-high subtype are susceptible to the development of a microenvironment inhibiting immune, which is distinguished by the upregulation of immune checkpoint expression and infiltration of immunosuppressive cells. This phenomenon may ultimately lead to an unfavorable prognosis.

### 3.5. Somatic mutations in 3 inflammatory subtypes

**Figure 5.**
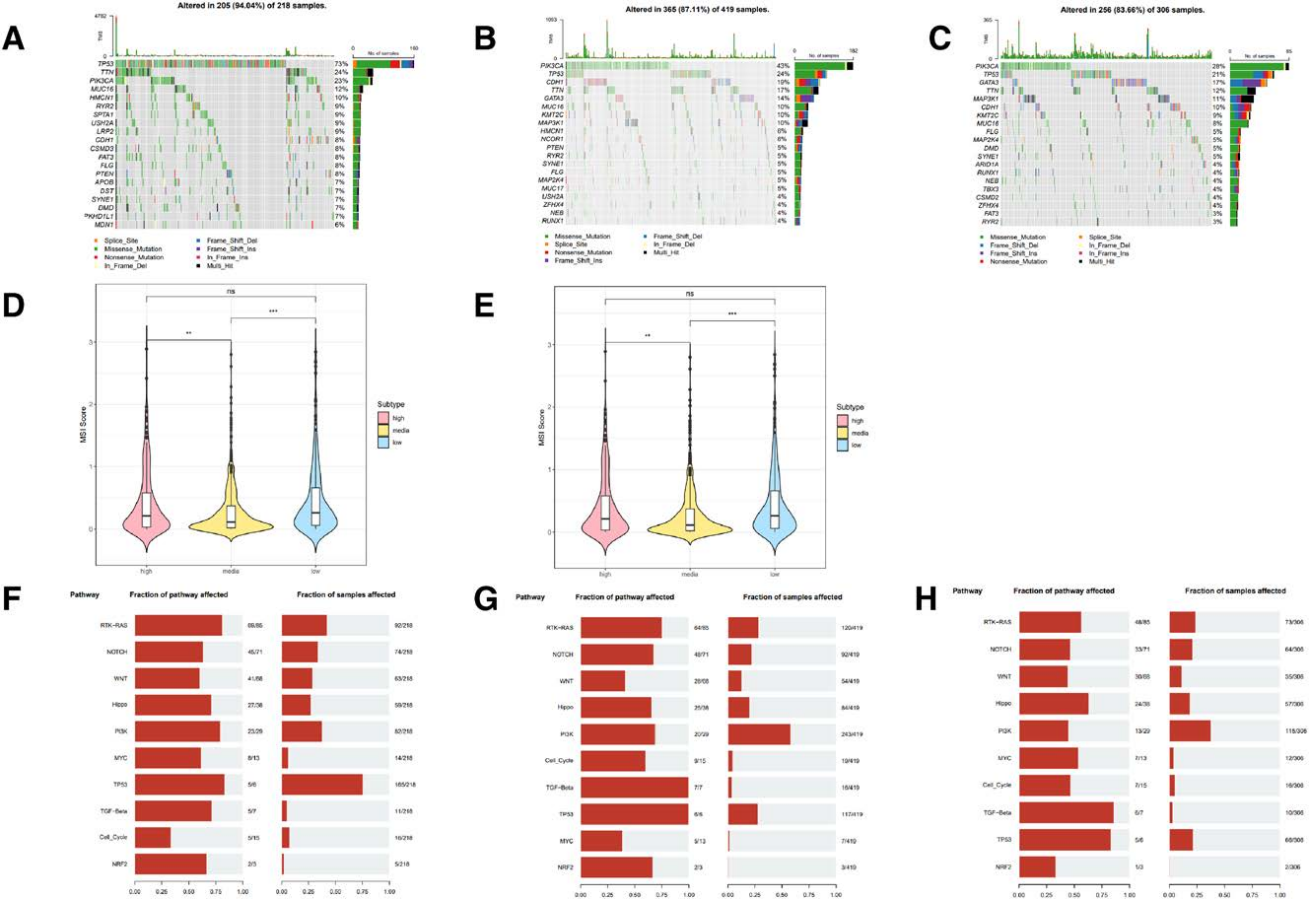
The analysis of somatic mutations across 3 subtypes of BRCA. (A–C) Waterfall plots depicting the most frequently mutated genes among the inflammation-high subtype (A), inflammation-media subtype (B), and inflammation-low subtype (C), encompassing the top 20 genes. (D) Violin plot showing the TMB score. (E) Violin plot representing MSI score in 3 subtypes. (F–H) The mutation frequencies of ten common oncogenic pathways. BRCA = breast cancer, TMBs = tumor mutation burdens.

Additionally, we observed unique somatic mutation patterns in these subtypes, with a high incidence of TP53 mutations in all 3 inflammatory subtypes. Notably, the highest mutation rate of TP53 was observed in the inflammation-high subtype, with rates of 73%, compared to rates of 24% and 21% in the middle- and low- inflammation subtypes, respectively. The data presented in Figure 5A–C indicated that CDH1 was found to be present in only 8% of the high inflammation response group, whereas it was present in 19% of the medium inflammation group and 10% of the low inflammation group. Additionally, the PIK3CA mutation rate was observed to be the highest in the inflammatory medium response group (43%), and the lowest in the inflammatory high response group (23%). Furthermore, Figure 5D indicated that the tumor mutation burden score of the subtype exhibiting high inflammation ranked first, whereas Figure 5E demonstrated that low inflammation exhibited a higher score in relation to microsatellite instability. TP53 and RTK-RAS were not evident in the low-inflammation group, although the results showed that many carcinogenic pathways were detectable in all 3 groups (Fig. 5F–H) (Fig. S2, Supplemental Digital Content, http://links.lww.com/MD/M494).

### 3.6. The establishment and validation of an inflammation-related prognostic signature

**Figure 6.**
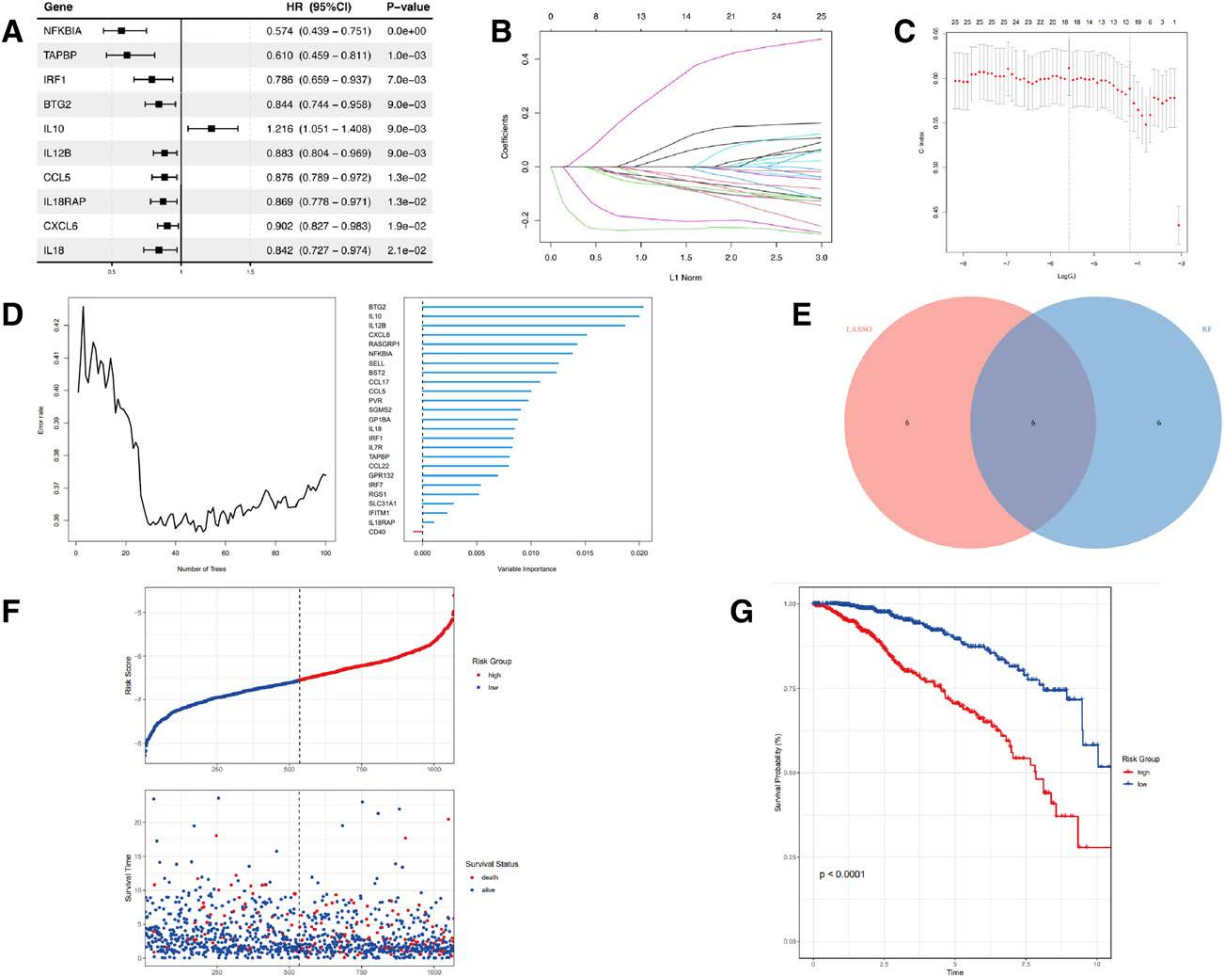
The creation and verification of a prognostic signature related to inflammation. (A) The top 10 genes selected from the Univariate Cox analysis presented. (B) Lasso Cox analysis revealed 12 genes exhibiting the strongest association with overall survival. (C) The analysis of the 12 genes examined by random forest analysis. (D) Hub genes by the interaction with Lasso Cox regression and Random Forest showed by Venn Diagram. (E) The distribution of risk scores and survival status of individual patient determined by utilizing 6 gene risk signatures. (F) Kaplan–Meier curves for patients categorized as either high- or low-risk based on their respective scores. G 3 testing cohorts from the GEO database (GSE 48390, GSE 58812, and GSE 103091).

The study employed a univariate Cox analysis in order to find 25 of 198 inflammatory genes that were significantly correlated with overall survival. Then the top ten genes with the most significant *P*-value were summarized in Figure 6A. 25 inflammation genes screened by univariate Cox proportional-hazards model analysis were selected for predicting the prognostic value of the model in the LASSO regression analysis and random forest analysis, respectively. Twelve genes were selected through LASSO regression analysis, and an additional 12 top-ranked genes were filtered by Random Forest analysis (Fig. 6B and C). The intersection of these 2 groups of genes resulted in 6 hub genes, as presented in the Venn diagram (Fig. 6D). Six hub genes were further evaluated in a multivariate Cox regression analysis. The risk-score model was constructed based on the following equation: RS = ∑_I_ Coefficient(mRNA) × Expression(mRNA_i_). The genes identified were NFKBIA, IL12B, CXCL6, IL10, BTG2, RASGRP1, respectively. Furthermore, an investigation was conducted to evaluate the association between the risk score and survival status. As depicted in Figure 6E, the count of surviving individuals in the low-risk group significantly increased when compared to that in the high-risk group. The predictive efficacy of the risk model was subsequently evaluated using Kaplan–Meier analysis (Fig. 6F). Finally, the validity of this model was tested using 3 cohorts from the GEO database. Three testing datasets are respectively GSE48390, GSE58812, and GSE 103091 (Fig. 6G). The findings demonstrated that the inflammation-related prognostic signature is both rational and significant.

### 3.7. The risk score signature exhibits noteworthy prognostic value

**Figure 7.**
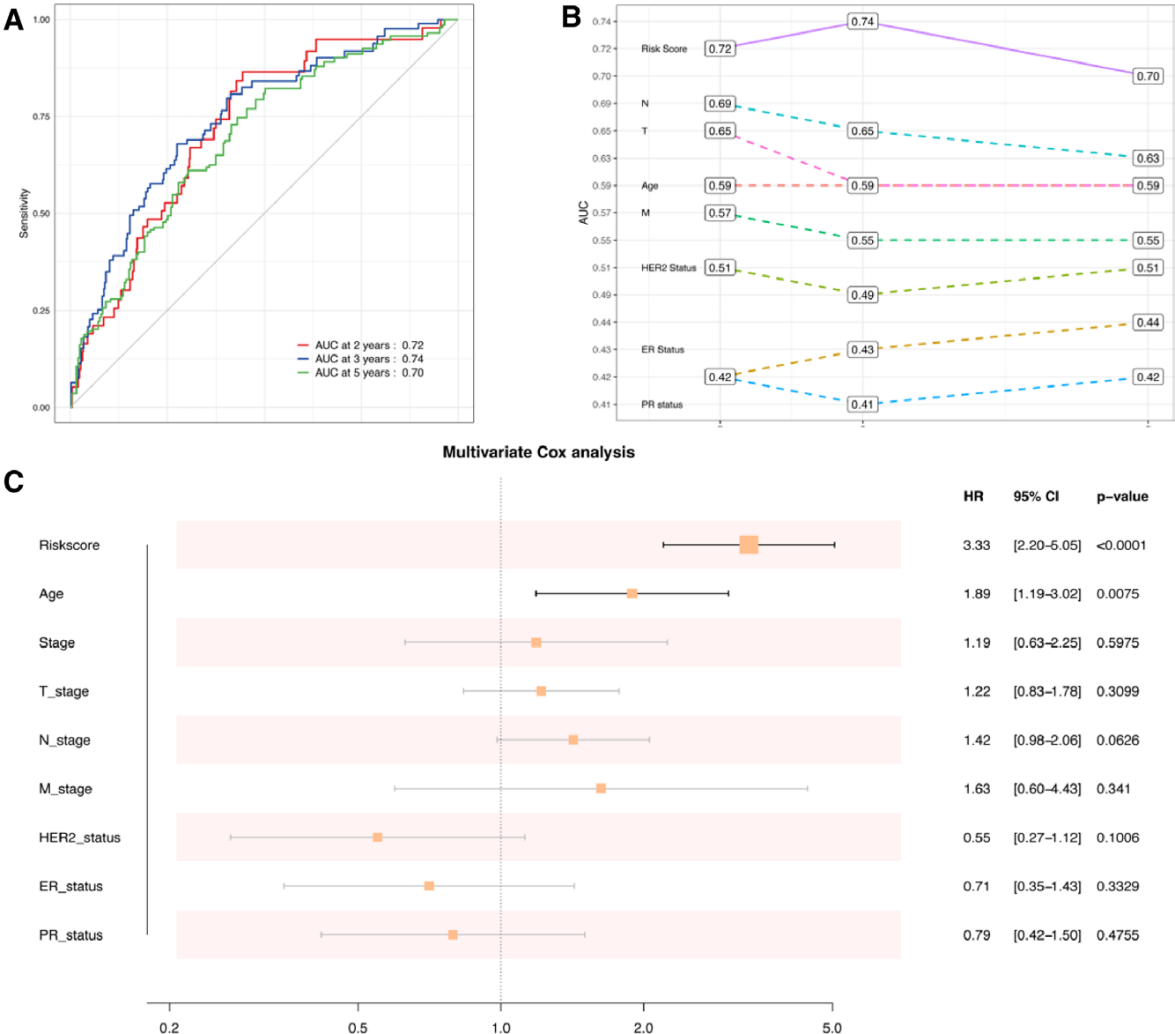
Risk prognostic signatures associated with inflammation. (A) ROC curve showing the survival rates of 2-, 3- and 5-years. (B) Comparative analysis of the predictive value between inflammation risk signature and clinical factors. (C) multivariate Cox regression analysis. ROC = receiver operating characteristic.

Based on survival rates over 2 years, 3 years, and 5 years, a ROC curve was used to assess the predictive effectiveness of the risk signature. The ROC curves, as measured by the area under the curve, yielded values of 0.72, 0.74, and 0.70, respectively, suggesting predictive power was high (Fig. 7A). Additionally, we evaluated the prognostic performance of the inflammation risk signature in BRCA based on clinical factors, including T, N, risk score, stage, and age.

The findings indicated that the risk score exhibited superior prognostic performance compared to other clinical characteristics, as evidenced by the results presented in Figure 7B. Furthermore, the multivariate analysis depicted in Figure 7C revealed that an evaluated risk score was independently associated with a significantly unfavorable overall survival, suggesting that it may be a reliable prognostic predictor for patients with BRCA.

### 3.8. Examining the relationship between the prognostic signature and chemosensitivity

**Figure 8.**
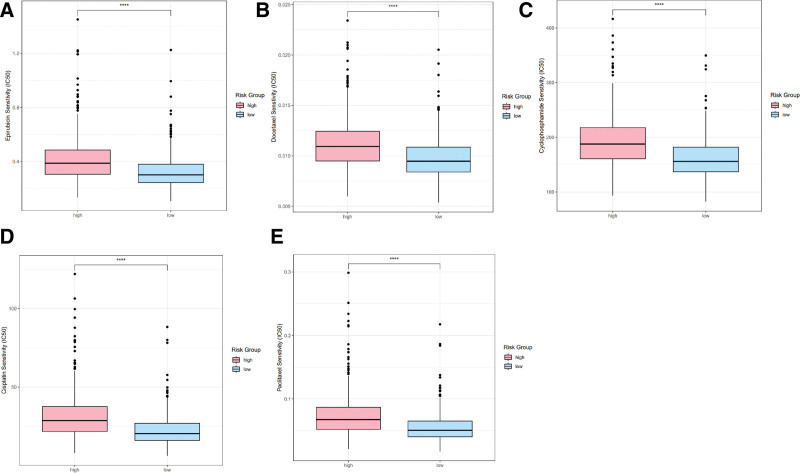
Boxplots showed the link between risk scores and patients’ sensitivity to chemotherapy drugs including epirubicin (A), docetaxel (B), cyclophosphamide (C), cisplatin (D), paclitaxel (E).

Chemotherapy is a crucial component of the sophisticated treatment regimen for BRCA. As such, our objective was to investigate if the risk scores could be linked to patients’ responsiveness to commonly utilized chemotherapeutic agents. Our findings indicate that widely prescribed medications, including epirubicin (*P* < .05, Fig. 8A), docetaxel (*P* < .05; Fig. 8B), cyclophosphamide (*P* < .05; Fig. 8C), cisplatin (*P* < .05; Fig. 8D), and paclitaxel (*P* < .05; Fig. 8E), exhibited higher IC50s values in the group with high risk. Consequently, this model presented considerable promise in forecasting chemosensitivity and could assist clinicians in selecting the optimal chemotherapy protocol.

### 3.9. Analyzing the effects of immunotherapy

**Figure 9.**
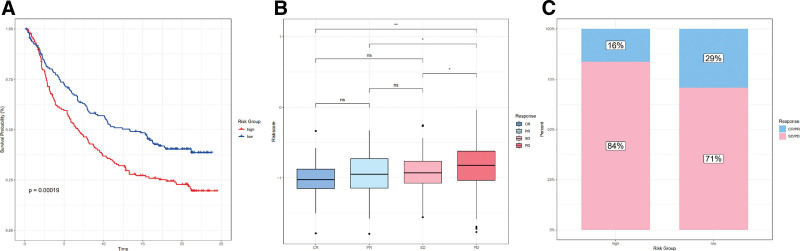
The role of the risk score signature in the immunotherapeutic responses. (A) Survival analysis for anti-PD-L1 clinical response between low- and high-risk groups. (B) Risk score showed in different anti-PD-L1 clinical response groups. (C) The percentage of patients responding to PD-L1 blockade therapy in low- and high-risk groups.

Immune checkpoint blockade is an excellent immunotherapy treatment for cancer that has been successful. In light of this, we investigated the potential of a risk score signature predicting the clinical response of patients undergoing immune checkpoint blockade therapy. Our analysis of the IMvigor210 cohort, which received anti-PD-L1 immunotherapy, revealed that patients with a low-risk score experienced a prolonged survival (*P* = .00019) (Fig. 9A). Additionally, patients who responded to treatment completely or partially had a lower risk score (Fig. 9B). Clinical responses to PD-L1 blockade therapy were improved in low-risk patients compared with high-risk patients (29% vs 16%) (Fig. 9C).

## 4. Discussion

Despite the widespread use of high-throughput tissue chip technology and the advancement of personalized treatment for breast cancer, the incidence and mortality rates of breast cancer keep rising annually, posing a significant threat to women’s health and well-being.^[29]^ The primary obstacle to achieving more effective diagnosis and treatment of drug targets is the scarcity of dependable and valuable biomarkers.

Our research identifies new molecular subtypes based on the inflammatory response, integrating somatic mutation analysis, immune infiltration, and the impact on chemotherapy and immunotherapy efficacy. Our study not only elucidates the distinctive inflammatory gene expression patterns across these subtypes but also establishes a prognostic signature that can significantly predict patient outcomes and therapeutic responses. Importantly, by delving into the interaction between inflammation-driven subtypes and the tumor microenvironment, we reveal potential pathways for targeted therapy that are not addressed by the existing literature. The comprehensive approach of assessing the tumor immune cycle and its association with somatic mutations within these subtypes further delineates the complex landscape of breast cancer, proposing a nuanced understanding that could lead to more personalized and effective treatment strategies. Thus, our findings contribute a significant layer of complexity to the understanding of breast cancer heterogeneity, offering a new angle on how inflammation influences prognosis and response to therapy, thereby underscoring the clinical value of our research in advancing personalized medicine in oncology.

Our study’s approach and findings resonate with and expand upon the themes explored in the resent study, yet we offer distinct insights and methodological advancements that underscore our contribution to the field of breast cancer research. Habanjar et al^[30]^ highlight the pivotal role of cytokines in shaping both pro-tumor and antitumor immune responses within the breast cancer microenvironment. Similar to our findings, they emphasize the dual nature of cytokines in breast cancer progression. However, our study extends beyond cytokine profiling to uncover novel molecular subtypes driven by the inflammatory response. By integrating somatic mutation and immune infiltration analysis, our research provides a more comprehensive understanding of the tumor microenvironment, thus offering refined targets for immunotherapy. Wang et al^[31]^ elucidate the transformative role of EGFR-targeted therapy in modulating the immunological landscape of the tumor microenvironment in inflammatory breast cancer. While their study focuses on the EGFR pathway’s impact on immune cell dynamics, our research broadens the scope to identify inflammatory-driven molecular subtypes across breast cancer. Our approach not only corroborates the importance of targeting key pathways like EGFR but also underscores the potential of utilizing inflammation-based classification for predicting response to combined modalities of targeted therapy and immunotherapy. Tkach et al^[32]^ reveal how extracellular vesicles from triple-negative breast cancer cells influence macrophage differentiation, leading to an immune microenvironment conducive to better clinical outcomes. This study’s focus on the role of extracellular vesicles complements our findings by demonstrating another layer of complexity in tumor-immune communication. Our identification of inflammation-based molecular subtypes adds to this narrative by showing how the broader inflammatory response, encompassing various sources including extracellular vesicles, correlates with patient prognosis and treatment response.

This study employed inflammatory responses to categorize BRCA into distinct groups, revealing that 3 subtypes with varying degrees of inflammation (high, medium, and low) exhibited diverse effects on TME, clinicopathological features, and prognosis. The results suggest that this classification system is both reasonable and clinically valuable. The subtype characterized by inflammation-high exhibited inferior survival prognosis performance, with the highest level of immune cell infiltration. Consequently, a prognosis model was constructed by integrating 6 hub genes. Subsequent testing of this model with datasets confirmed its high value. Additionally, a drug sensitivity analysis was performed on both high- and low-risk groups, revealing that the high-risk group demonstrated greater sensitivity to chemotherapy drugs. Furthermore, high-risk patients were found to be relatively less successful with PD-L1 blocking therapy than low-risk patients. Several scholarly articles have highlighted the significant contribution of the tumor microenvironment (TME) in the progression and metastasis of breast cancer.^[33]^ The TME of breast cancer is mainly composed of immune cells, tumor cells, and stromal cells. Immune cells contained in breast cancer TME include dendritic cells, regulatory T cells, T lymphocytes, and macrophages.^[34]^ These immune cells also work together to promote the regulation of the tumor microenvironment.

The regulation of the tumor microenvironment is typically attributed to immune cells exhibiting immunosuppressive phenotypes, including tolerogenic dendritic cells, regulatory T cells, and tumor-associated macrophages.^[35]^ It is widely recognized that macrophages can generate mutagenic inflammatory microenvironments that facilitate angiogenesis and enable cancer cells to evade immune surveillance.^[36]^ Consequently, an increasing body of research has directed attention towards macrophages as a potential target for checkpoint inhibitors.^[37]^ MDSCs are a heterogeneous population of immature cells that originate from bone marrow progenitor cells. These cells typically suppress the immune response, thereby promoting tumor growth through the inhibition of T cell proliferation and interaction with the PD-1 receptor.^[38,39]^ Specifically, the main mechanism is Treg lymphocytes allow cancer immune escape in tumors and lead to faster development of cancer.^[40]^ Consequently, targeting Treg cells represents a promising avenue for the development of novel cancer immunotherapies.^[41]^

Our findings aligned with the aforementioned researches’ conclusion that patients with varying inflammatory subtypes exhibit distinct tumor microenvironments and immune cell infiltration patterns. Specifically, our study revealed a notable prevalence of MDSCs, Regulatory T cells, and Macrophages in the inflammation-high subtype. Moreover, we determined that patients categorized under the inflammation-high group are at a heightened risk of experiencing unfavorable prognoses due to the presence of immunosuppressive microenvironments characterized by elevated immune checkpoint expression and immunosuppressive cell infiltration.

Nowadays, the utilization of bioinformatics analyses and machine learning algorithms to discover novel gene prognostic signatures is a prominent area of research.^[42]^ Within the context of breast cancer, numerous prognostic measures and treatments rely on the expression of pivotal genes, with ER, PR, and HER2 serving as the most employed indicators in clinical practice.^[43]^ The treatment of breast cancer has evolved from a singular surgical intervention to personalized adjuvant therapy, such as chemoradiotherapy.^[44–46]^

According to recent guidelines, the administration of adjuvant chemotherapy drugs targeting ER, PR, and HER-2 has been shown to significantly enhance the survival rates of individuals with breast cancer.^[47,48]^ It is widely acknowledged that multi-omic analysis surpasses single-gene expression analysis in terms of efficacy. Therefore, in comparison to other studies, the utilization of Risk Score as a multi-gene signature, which comprises 6 key inflammatory genes, is deemed more compelling and consistent. Also, in this study, the expression of genes in prognostic signature were tested by high-throughput sequencing and this technology is widely acknowledged that it can be more accurate. In addition, immunomodulator therapies are mainly about anti-PD-L1 and PD-L1 antibodies.^[49]^ In the case of anti-PD-1 and PD-L1 antibody therapy, the binding of PD-1 to its ligand PD-L1 weakens the activity of immunoregulatory T cells.^[50,51]^ The application of blockers can restore immune activity in the tumor microenvironment and promote the maturation of DC cells and enhance the anti-tumor immune response.^[52]^ In the future, this new signature can distinguish and evaluate the effect of these drugs, which greatly improves drug selection and maximum efficacy.

However, this study has certain constraints, such as a relatively limited sample size compared with other papers. Secondly, while our bioinformatic analysis suggests that inflammation classification may be a novel approach to breast cancer treatment, the conclusion has yet to be validated via in vitro or in vivo experiments. Thirdly, the efficacy of immune checkpoint inhibitors in triple-negative breast cancer has not been subjected to specialized analyses. Given the higher incidence of PD-L1 positivity in triple-negative breast cancer, clinical trials have predominantly focused on this patient population.^[53–55]^

## 5. Conclusion

In conclusion, the novel classification system predicated on the inflammatory response of breast cancer holds immense importance in the realms of diagnosis, treatment, drug selection, and prognosis. Also, the Risk Score is an independent predictor for breast cancer.

## Acknowledgments

We thank the investigators and research groups of the GSE48390, GSE58812, GSE103091, IMvigor210, and TCGA datasets who participated in the collection of specimens and shared the information publicly.

## Author contributions

**Conceptualization:** Ke Yu.

**Data curation:** Ke Yu.

**Investigation:** Chi Xu.

**Methodology:** Ke Yu, Chi Xu.

**Resources:** Feng Wang.

**Software:** Ke Yu, Feng Wang.

**Supervision:** Hua Wang.

**Visualization:** Hua Wang.

**Writing – original draft:** Ke Yu.

**Writing – review & editing:** Hua Wang.

## Supplementary Material

**Figure:**
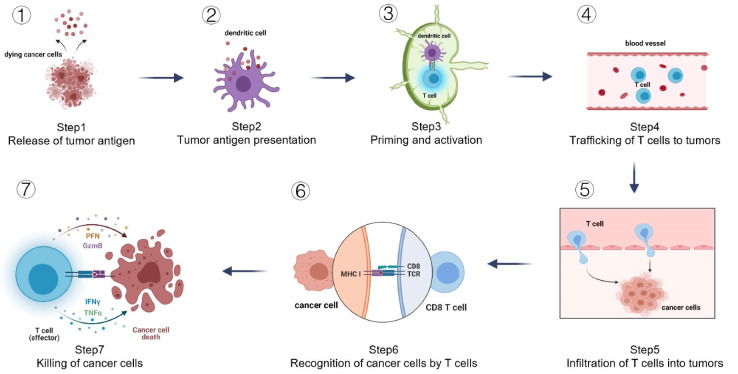


**Figure:**